# A case of pheochromocytoma presenting with cardiac manifestation: case report

**DOI:** 10.1186/s12887-020-02197-4

**Published:** 2020-06-17

**Authors:** Akbar Molaei, Vahideh Abarzadeh-Bairami, Seyyed-Reza Sadat-Ebrahimi

**Affiliations:** 1 Cardiovascular Research Center, Shahid Madani Heart Center, Tabriz University of Medical Sciences, Tabriz, Iran; 2 Pediatric Health Research Center, Tabriz Children Hospital, Tabriz University of Medical Sciences, Tabriz, Iran

**Keywords:** Pheochromocytoma, Cardiomyopathy, Hypertension, Cardiac involvement, Case report

## Abstract

**Background:**

Pheochromocytomas are rare tumors originating in chromaffin cells which predominantly are located in adrenal glands. Sustained or paroxysmal hypertension (HT) is the most frequent sign of pheochromocytoma. In some cases, it is associated with the classic triad including episodic headaches, sudoresis, and tachycardia; however, we present a case of pheochromocytoma with first presentation of cardiomyopathy.

**Case presentation:**

The authors describe a rare case of a pheochromocytoma which was first presented with cardiomyopathy in a 7-year-old patient. The patient was admitted with malaise, abdominal pain, polydipsia, and myalgia. Further evaluations revealed hyperglycemia, mild dehydration and sinus tachycardia but no HT. Echocardiography demonstrated some of the signs of cardiomyopathy which was incorrectly diagnosed as viral myocarditis. The patient was discharged with this diagnosis but he presented again with HT crisis a few months later. A diagnosis of pheochromocytoma was assigned after the evaluation of the HT secondary causes. The diagnosis was confirmed by metanephrine assay and the tumor was localized in the adrenal gland using the abdominal MRI.

**Conclusion:**

Pheochromocytoma can present itself with normotensive cardiomyopathy. Therefore, the possibility of pheochromocytoma should be considered in patients with cardiomyopathy especially in those with positive familial history.

## Background

Pheochromocytoma is a rare neuroendocrine tumor that produces catecholamines and other neuropeptides, originating mainly in the adrenal gland medulla. It has an annual incidence of approximately 0.8 per 100,000 person-years [1]. The majority of cases are sporadic, but 10–25% of the cases can be associated with genetic syndromes such as Von Hippel-Landau (VHL) disease, type 1 neurofibromatosis and multiple endocrine neoplasia type 2 (MEN 2) [2]. Sustained or paroxysmal hypertension (HT) is the most frequent sign of pheochromocytoma. In some cases, it is associated with the classic triad including episodic headaches, sudoresis, and tachycardia [3]. Cardiovascular complications due to adrenergic stimulation can potentially be fatal, emphasizing the importance of timely diagnosis and effective therapeutic strategy. In the current case report, we describe a rare case of a pheochromocytoma which was first presented with cardiomyopathy in a 7-year-old patient. The case is reported after obtaining the permission of the institutional review board.

## Case presentation

A 7-year-old boy with a negative history for any particular diseases presented with malaise and abdominal pain to the emergency room. These symptoms had appeared since 1 month before admission and were gradually worsen in the last 2 weeks. The patient had also developed polydipsia and myalgia. On admission, the patient was afebrile (body temperature [BT], 36.7), with a pulse rate (PR) of 200/min [150/min after hydration], a respiratory rate of 25/min, blood pressure (BP) of 105/65 mmHg, and blood sugar (BS) of 256 mg/ml. Oral mucosa was dehydrated but other examinations revealed no significant findings. Laboratory test results are reported in Table 1. The patient was admitted to endocrinology service with the diagnosis of new-onset diabetes mellitus type 1 (DM1). BS was controlled (fasting BS = 100–110 mg/dl, postprandial BS = 115–140 mg/dl) by administration of insulin. Electrocardiogram showed normal sinus tachycardia. On the transthoracic echocardiography, there was a mild pericardial effusion, left ventricular (LV) ejection fraction (EF) of 30–35%, mild LV hypertrophy and LV diameter of 33 mm. Considering sinus tachycardia, echocardiography findings, white blood cell count (WBC) of 17,200/μl, and erythrocyte sedimentation rate (ESR) of 60, the cardiologist suggested a diagnosis of cardiomyopathy due to viral myocarditis. Treatment with dopamine (1 μg/kg), milrinone (50 μg/kg stat and 5 μg/h infusion), furosemide (1 mg/kg two times daily), captopril (6.25 mg two times daily), and carvedilol (6.25 mg two times daily) was initiated and insulin therapy was continued with the same dose. BP monitoring was conducted but the patient was normotensive. PR was gradually reduced from 140 to 150/min to 105–100/min in a few days.. WBC was decreased from 17,200 to 10,940/μl (Table 1). The patient was discharged and the following medications were prescribed to be taken at home: captopril (12.5 mg every 12 h), carvedilol (6.25 mg every 12 h), insulin glargine (4 units each night) and insulin aspart (2 units before each meal). In the outpatient visits, the patient had adequate BS control, normal BP, and was asymptomatic except for a mild malaise and inadequate weight gain.

**Table 1:** Laboratory tests results at first admission

Laboratory tests	Time	
	Result in admission	Result before discharge
**Urine**		
WBC	2–3	
RBC	1–2	
Bacteria	Negative	
Glucose	+++	
Crystal amurate	Many	
Ketone	Negative	
**Blood (serum)**		
WBC (1000/mm^3^)	17.2	10.9
Lmyph (%)	34.4	34.5
Neut (%)	58.8	54.7
Hb (g/dl)	13.3	12.4
Plt (1000/mm^3^)	561	510
BS (mg/dl)	180	
Urea (mg/dl)	25	
Creatinine (mg/dl)	0.6	
Na (mg/dl)	136	
K (mg/dl)	4.2	
ESR	60	60
C-reactive protein	++	
Blood culture	Negative (in three times repetitions)	
**Venus blood gas analysis**		
PH	7.48	
PCO2	25.6	
PO2	66.4	
HCO3	18.8	

*WBC* white blood cells count, *RBC* Red blood cells count, *Hb* Hemoglobin, *ESR* Erythrocyte sedimentation rate

After 21 months the patient was admitted again due to HT (BP, 189/140 mmHg), nausea, sudoresis, and malaise. No headache, tremor, pallor, dyspnea, and generalized weakness were seen in the patient before admission. The BP was reduced to 140/80 mmHg and transferred to ICU. The patient developed a headache during his stay in ICU.

**Fig. 1:**
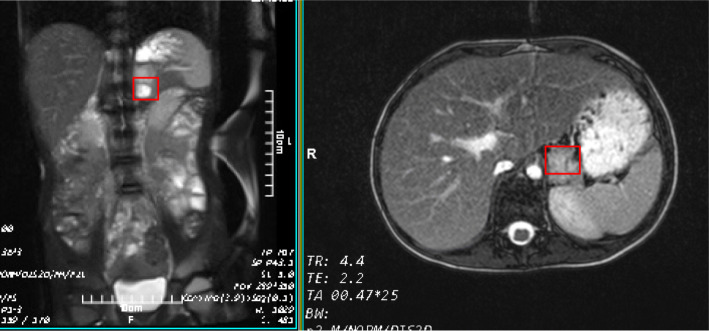
Abdominal magnetic resonance imaging (MRI) which depicts a left adrenal gland nodule (red boxes, over 3 cm diameter)

**Fig. 2:**
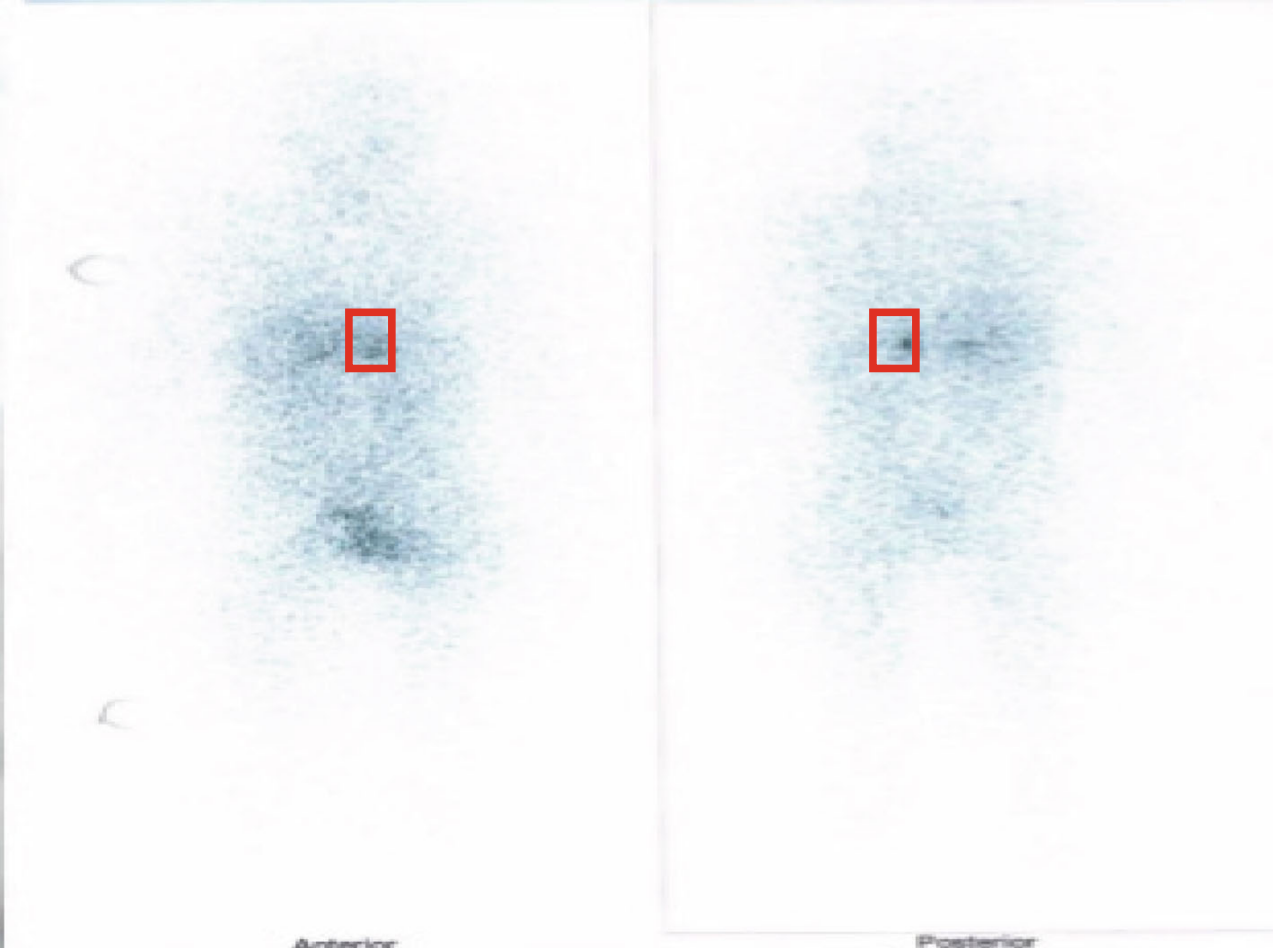
Bilateral MIBG avid areas in both adrenal glands

**Fig. 3:**
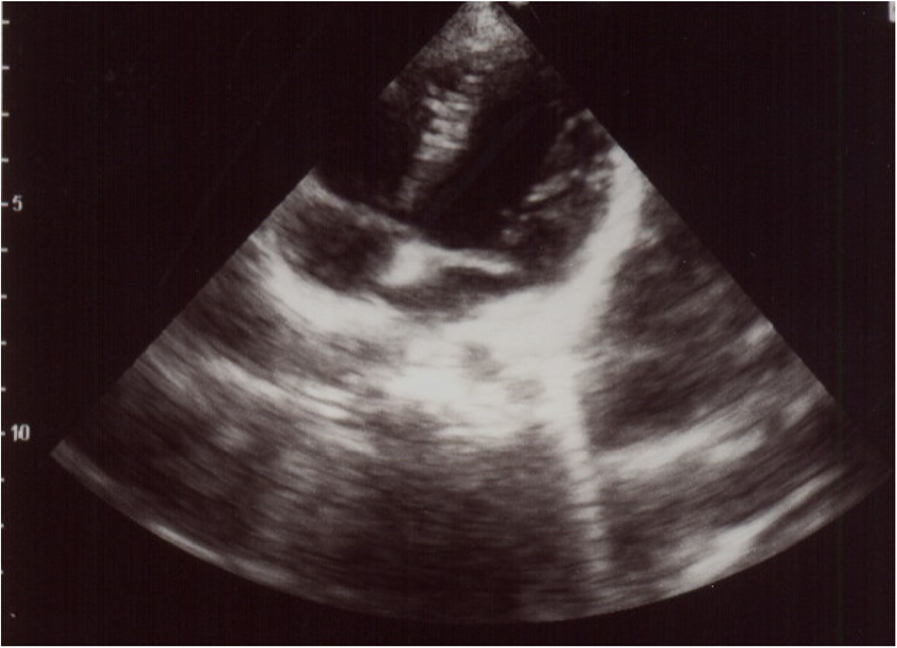
Transthoracic echocardiography revealed normal four-chamber size and function, except for a mild LVH and mild tricuspid regurgitating with an LVEF of 55%

Doppler ultrasonography of renal veins and arteries was performed but no significant pathologies were detected. Considering the positive familial history of pheochromocytoma in his uncle and clinical suspicion for pheochromocytoma, the urinary levels of metanephrine, epinephrine, norepinephrine, and vanillylmandelic acid were assessed. Hormonal assays revealed elevated serum and urinary normetanephrine, norepinephrine, and metanephrines (Table 2). Furthermore, a metaiodobenzylguanidine (MIBG) scan and abdominal MRI were performed. Abdominal MRI confirmed the presence of a 35 × 25 × 16 mm mass at the left adrenal gland, which was hyper-signal on T2WI and hypo-signal on T1WI. The right adrenal gland was unremarkable (Fig. 1). MIBG scintigraphy identified bilateral uptake increase in the regions of adrenal glands. Normal uptake was seen in other parts of the body. Bilateral MIBG avid areas in both adrenal glands were suggestive for pheochromocytoma (Fig. 2). In transthoracic echocardiography, a mild LVH and a mild tricuspid regurgitating were reported. LVEF was 55% (Fig. 3).

**Table 2:** Laboratory tests results at second admission

Laboratory tests	Result	Reference value
**Urine**		
Urinary Norepinephrine (μg/24 h)	490	< 90
Urinary Epinephrine (μg/24 h)	13.14	< 20
Urinary metanephrines (μg/24 h)	58	25–312
Urinary normetanephrines (μg/24 h)	1133.2	< 600
WBC	1–2	< 5
RBC	0–1	0–1
Bacteria	Negative	
Crystal amurate	Few	
Glucose	Negative	
Protein	Negative	
**Blood (serum)**		
Cortisol (μg/dl)	18.2	4.5–25.0
Renin (mlU/ml)	> 500	2.8–39.9 (supine posture)
Aldosterone (ng/dl)	62.8	3.7–31 (supine posture)
TSH (mlU/L)	4.2	0.4–6.21
FT4 (ng/dl)	9.5	6.4–15

*WBC* white blood cells count, *RBC* Red blood cells count, *TSH* Thyroid releasing hormone, *FT4* Free thyroxine

Treatment with oral Ca channel blocker, beta-blockers was initiated (amlodipine 5 mg and metoprolol 50 mg) and followed by losartan (25 mg daily) to adequately normalize BP.

The left side adrenal tumor was removed by surgery and the right side was examined but remained intact. Histopathological evaluations confirmed the diagnosis of pheochromocytoma. After surgery, the patient’s medications were tapered and discontinued. He remained normotensive and BS was in a normal range. The symptoms faded away and the patient was discharged with no further medications but was advised to return regularly for outpatient visits.

During 1 year follow-up after discharge, the patient remained asymptomatic. He was not receiving any medications. The BS was in the normal range and the patient was normotensive.

## Discussion

We described a clinical case of a 7-year-old boy with the first presentation of cardiomyopathy due to pheochromocytoma. Pheochromocytoma is symptomatic in nearly half of the patients. These symptoms are typically paroxysmal. One-half of the symptomatic patients have paroxysmal hypertension; however, nearly 5 to 15% of patients present with normal BP [1, 4]. As a very rare incidence, pheochromocytoma is associated with cardiomyopathy. This phenomenon is ascribed to the excessive release of catecholamines mainly epinephrine and norepinephrine, which stimulate adrenergic receptors. It occurs with similar pathophysiology to that in stress-induced (takotsubo) cardiomyopathy [5]. These patients are reported to present with pulmonary edema; however, no pulmonary edema was detected in our patient [6]. It is suggested to perform echocardiography for symptomatic patients. Although, it is usually normal in asymptomatic patients, including those with asymptomatic hypertension [7]. A study on 26 consecutive patients with pheochromocytoma reported that echocardiographic evaluations in 62.1% of the patients were normal but 27.6% of the patients had concentric LV hypertrophy with normal LV systolic function and 10.3% had LV systolic dysfunction. In only three symptomatic patients, echocardiography revealed catecholamine cardiomyopathy with transient LV dysfunction [8]. Our patient in the first admission was diagnosed as cardiomyopathy due to viral myocarditis. This diagnosis could explain most of the patient’s signs and symptoms. The clinical presentations of myocarditis are greatly variable ranging from subclinical disease, to fatigue, chest pain, arrhythmias, heart failure, cardiogenic shock, and sudden death. Sinus tachycardia is the most common arrhythmia in patients with myocarditis which was also present in our patient. Moreover, our patient’s echocardiographic findings including LV dilatation without hypertension, systolic dysfunction, and mild pericardial effusion were all suggestive of myocarditis. Although, this diagnosis could explain most of our observations in this patient, we understood that pheochromocytoma as an important differential diagnosis should have been considered in this case. Furthermore, at the first admission, our patient presented an abnormal glucose level which was diagnosed incorrectly as DM1, though, it was a carbohydrate metabolism impairment that was directly related to the catecholamine excess due to pheochromocytoma.

The exact mechanism of cardiomyopathy despite normal blood pressure in pheochromocytoma patients is not clearly explained in the literature. We assumed that possibly undetected transient hypertension crisis had induced cardiac failure and dilatation in this patient; however, the drop in noradrenaline secretion and heart failure have made that we only detect normal blood pressure between the episodes of hypertension crisis.

The first presentation of this patient was in some way ambiguous and challenging. However, the symptoms in the second presentation were more suggestive of pheochromocytoma in particular hypertension. Considering the age of our patient, the most probable etiologies for hypertension would be renal/renovascular, endocrine diseases or aortic coarctation. Renal/renovascular etiologies are the most common in this age; however, the absence of abdominal murmurs, normal renal function and Doppler ultrasonography of the renal arteries rolled out such diagnosis. Aortic coarctation was rolled out using transthoracic echocardiography. Therefore, pheochromocytoma remained the most probable endocrine disease which may cause hypertension in this case. Our patient had elevated renin and aldosterone levels. This phenomenon is reported to occur in some patients with pheochromocytoma [9, 10].

After clinical suspicion of a pheochromocytoma, tumor localization is essential with proper imaging such as abdominal CT or MRI and I131-MIBG scintigraphy. Approximately 85 to 90% of catecholamine releasing tumors are intra-adrenal but about 10 to 15% of them are extra-adrenal and are referred to as catecholamine-secreting paragangliomas. Different therapeutic options are available for each category. Though, the best choice for our patient was surgery after BP normalization with Ca Blocker and B-adrenergic drugs. Following resection of catecholamine secreting mass, the patients became asymptomatic and the laboratory tests were normal. Moreover, no further medication was indicated.

Long-term follow-up is required for all patients with pheochromocytoma due to the possibility of recurrence. Considering the dramatic response of our patient to treatment and normal results of medical examinations and laboratory tests until 1 year, he has apparently a favorable prognosis; however, the possibility of tumor relapse still necessities long-term follow-up [11].

## Conclusion

Pheochromocytoma is one of the secondary causes of HT that can be treated surgically. However, it can present itself with normotensive cardiomyopathy. Therefore, the possibility of pheochromocytoma should be considered in patients with cardiomyopathy especially in those with positive familial history.

## Data Availability

All Data and material collected during this study are available from the corresponding author upon reasonable request.

## Abbreviations

HT: Hypertension
VHL: Von Hippel-Landau disease
MEN 2: Multiple endocrine neoplasia type 2
BT: Body temperature
PR: Pulse rate
BP: Blood pressure
BS: Blood sugar
DM1: Mellitus type 1
LV: Left ventricular
EF: Ejection fraction
WBC: White blood cell count
ESR: Erythrocyte sedimentation rate
ICU: Intensive care unit
MIBG: Metaiodobenzylguanidine
